# Factors Associated with* Klebsiella* Bacteremia and Its Outcome in Under-Five Children Admitted with Diarrhea

**DOI:** 10.1155/2016/4760610

**Published:** 2016-08-29

**Authors:** Shamima Akhter, Tahmeed Ahmed, Shafiqul Alam Sarker, Monira Sarmin, Abu S. M. S. B. Shahid, K. M. Shahunja, Shoeb Bin Islam, Lubaba Shahrin, Tahmina Alam, Nur Haque Alam, Mohammod Jobayer Chisti

**Affiliations:** Nutrition and Clinical Services Division (NCSD), International Centre for Diarrhoeal Disease Research, Bangladesh (icddr,b), Dhaka 1212, Bangladesh

## Abstract

Although* Klebsiella* bacteremia in children is perceived to be associated with fatal consequences, data are scarce on those children presenting with diarrhea. We evaluated the factors associated with* Klebsiella* bacteremia in such children. In this retrospective chart analysis, data of all diarrheal children was retrieved from electronic medical record system (named as SHEBA) of Dhaka Hospital of International Centre for Diarrhoeal Disease Research, Bangladesh (icddr,b), from January 1, 2010, to December 31, 2012, who had their blood culture done. This was a study having a case-control design where comparison of clinical and laboratory characteristics was done among children with* Klebsiella* bacteremia (cases = 30) and those without any bacteraemia (controls = 90). Controls were selected randomly. The cases more often had fatal outcome (*p* < 0.001). In logistic regression analysis, after adjusting for potential confounders such as young age, severe dehydration, severe wasting, abnormal mentation, hypotension, and fast breathing, the cases were independently associated with hospital-acquired infection and positive stool growth (for all, *p* < 0.05). The study highlights the importance of obtaining blood cultures in hospitalized children under five years old with diarrheal illness in the presence of either hospital-acquired infection or positive stool culture to have better outcome.

## 1. Introduction

Diarrhea still remains as one of the leading killer diseases of children under five in developing countries and accounts for 9% of 6.3 million global deaths in 2013 [1]. Death is even higher in diarrheal children having bacteremia compared to those without [2]. An earlier report [3] from icddr,b has shown factors that were associated with an increased risk of death in bacteremic patients who were infected with a Gram-negative pathogen; however, among the Gram-negative bacteremia,* Klebsiella* is one of the most virulent pathogens and is often associated with high morbidity and mortality in children [3]. It has also been found to be the most common cause of pneumonia in severely malnourished children [4].* Klebsiella* bacteremia is perceived to be more common in diarrheal children compared to those without diarrhea and often have fatal outcomes. In resource-poor settings, where laboratory facilities are limited and blood culture is seldom done, clinical features may help in predicting* Klebsiella* bacteremia in such children. However, to our knowledge, there is no published data on the role of* Klebsiella* bacteremia and its clinical features in diarrheal children. The objective of our study was to evaluate the factors associated with* Klebsiella* bacteremia in under-five diarrheal children and their outcome. 

## 2. Materials and Methods

### 2.1. Ethical Statement

This study was solely a medical record analysis. This study did not involve any interviews with patients or care givers. Data were anonymous before analysis.

### 2.2. Study Site

The study population was treated in the Dhaka Hospital of International Centre for Diarrhoeal Disease Research, Bangladesh (icddr,b) and the description of the study site has been provided elsewhere [5].

### 2.3. Study Design

All the diarrheal children under five who had their blood culture done between January 1, 2010, and December 31, 2012, were enrolled in the study. A case-control design was deployed in the study where the children having* Klebsiella *bacteremia constituted the cases and those without any bacteremia constituted the controls. Thus, we did not include the children with positive blood cultures for organisms other than Klebsiella species as potential controls. We had taken threefold controls using randomization process in the Statistical Package for Social Science (SPSS) to increase the power of our analysis.

### 2.4. Patient Management

Management of the patients has been done following standardized protocol followed in the Dhaka Hospital of icddr,b and the management of hospitalized patient has also been described elsewhere [5].

### 2.5. Data Collection and Measurements

Data of all diarrheal children was retrieved from electronic medical record system (named as SHEBA) of Dhaka Hospital. After admission, every patient received a unique identification number. All the data were recorded under this number. Case report forms (CRF) were developed for this study, pretested, and finalised for data acquisition. Characteristics analyzed included demographics (age, gender), nutritional status including severe wasting (*z* score for weight for length/height < −3 of WHO growth standard) and severe underweight (*z* score for weight for age < −3 of WHO growth standard), abnormal mentation (drowsiness, convulsion, or restlessness), fast breathing (<2 months: ≥60/min; 2–<12 months: ≥50/min; 12–59 months: ≥40/min), SpO_2_ (transcutaneously measured blood oxygen concentration), dehydration status, hypotension (defined as systolic blood pressure ≤ 70 mm of Hg or diastolic blood pressure ≤ 40 mm of Hg or mean arterial pressure ≤ 50 mm of Hg) [6], hospital-acquired infection (new episode of infection at least after 48 hours of hospitalization), laboratory investigation (creatinine in micro-mol/L, stool culture for* Vibrio cholerae*,* Shigella* species,* Salmonella typhi*, and other vibrios), and outcome (deaths).

### 2.6. Statistical Analysis

All data were entered into a personal computer and edited before analysis using SPSS for Windows (version 17.0; SPSS Inc., Chicago, IL, USA) and Epi Info (version 6.0; USD, Stone Mountain, GA, USA). Differences in proportions were compared by the Chi-square test. In normally distributed data, differences in means were compared by Student's *t*-test, and the Mann-Whitney test was used for comparing data that were not normally distributed. A probability of less than 0.05 was considered statistically significant. Strength of association was determined by calculating odds ratio (OR) and their 95% confidence intervals (CIs). To identify clinical predictors associated with* Klebsiella* bacteremia in diarrheal children, variables were initially analyzed in a univariate model, and then, after adjusting for potential confounders, a multiple logistic regression model was used to identify the independent predictors of* Klebsiella* bacteremia.

## 3. Results

During the 3-year study period, a total of 3313 children fulfilled the study criteria and we only identified 30 (0.9%) cases of* Klebsiella* bacteremia. Among the remaining 3283 children, 676 had bacterial isolates other than* Klebsiella species.* Thus, we had randomly selected 90 controls among a total of 2607 children who had no growths in their blood culture and were available for the selection of the controls. Diarrheal children with* Klebsiella *bacteremia more often presented at their infancy, received antibiotics prior to admission, and had severe dehydration, fever, abnormal mentation, severe wasting, and positive stool growth on admission and they frequently developed hospital-acquired infection and more often had fatal outcome during hospitalization compared to those without bacteremia (Table 1). Other variables in Table 1 were comparable among the groups. In logistic regression analysis, after adjusting for potential confounders such as young age, severe dehydration, severe wasting, abnormal mentation, hypotension, and fast breathing, the cases were independently associated with hospital-acquired infection and positive stool growth (Table 2). Importantly,* Klebsiella* species causing bacteremia was found to have higher resistant (68%) to ceftriaxone. Bacterial isolates from stool have been shown in Table 3.

## 4. Discussion

Gram-negative bacteremia is a major cause of morbidity and mortality if undiagnosed on time [7, 8]. The observation of higher case fatality rate in children with* Klebsiella* bacteremia compared to those without any bacteremia was expected. We are not aware of any report on mortality due to* Klebsiella* bacteremia in diarrheal children. However, a number of previous studies in nondiarrheal children revealed that children with Gram-negative bacteremia more often had fatal outcomes compared to those without bacteremia [7, 8].

In this study, we observed that the prevalence of* Klebsiella* bacteremia is very low in children with diarrhea. However, in a number of hospital based studies, a higher prevalence was demonstrated that ranged from 13% [8] to 26% [9]. In a study from Ghana, 26%* Klebsiella* bacteremia was detected in children below 1 month of age [9]. Also in the same age group reports from Nigeria, Ghana, and the United States,* Klebsiella *accounts for 3 to 7% of all nosocomial bacterial infections [10]. The potential reasons for variations in prevalence of* Klebsiella* bacteremia might be due to heterogeneity in geographical location, patient population, study design, or differences in prior antibiotic therapy. Moreover, all of our study children had diarrhea which was not present in those above-mentioned studies [10] and that might be one of the reasons of variation in prevalence of* Klebsiella* bacteremia in our study children. To our knowledge this is the first study that reported the data on* Klebsiella* bacteremia in under-five diarrheal children from Bangladesh. One of the plausible reasons for the development of bacteremia in diarrheal children might be due to bacterial translocation through transcellular and paracellular pathways. It mostly occurs in immune compromised children and impairment of normal ecological balance of gut, mucosal barrier permeability, and stress [11].

In this study, we found an independent association of hospital-acquired infection and positive stool growth with* Klebsiella* bacteremia. On the other hand, young age, severe dehydration, severe wasting, and abnormal mentation were associated with* Klebsiella* bacteremia in a univariate analysis, although, in logistic regression analysis, their association with* Klebsiella* bacteremia became insignificant. While hypotension and fast breathing are the components of sepsis in adults following qSOFA criteria [12] and both in adults and children following Surviving Sepsis Guidelines [13], these two variables did not have significant association with* Klebsiella* bacteremia in our study children either by a univariate analysis or a logistic regression.

The US National Healthcare Safety Network indicates that infection due to Gram-negative bacteria is responsible for more than 30% of hospital-acquired infections.* Klebsiella* was found to be very common organism responsible for such type of infection in children [14].

To our knowledge, the observation of an independent association of* Klebsiella *bacteremia with bacterial isolates from diarrheal stool in our study has been described for the first time in medical literature. We do not have any ready explanation for this observation but altered gut function, mucosal damage, enteropathy, or absence of antibacterial peptide or loss of bacterial flora in diarrhea may have influences on translocation of* Klebsiella* from the gut [11, 15]. We have also evaluated sensitivity for extended spectrum beta-lactam (ESBL) antibiotics among* Klebsiella* species and in our reference laboratory, the surrogate marker of ESBL is ceftriaxone;* Klebsiella* species was found to have higher resistance (68%) to ceftriaxone. The finding is consistent with previous observation [16].

Gram-negative bacteremia is relatively common in young age and occurs much less frequently after the first year of life. Infants had the highest incidence of Gram-negative bacteremia among all children observed in a previous study [17], which is consistent with our study and* Klebsiella *was one of the commonest organisms isolated [17]. The increased incidence of* Klebsiella *bacteremia in young age might be due to less cellular and humoral immunity.

Wasting is an important indicator of acute malnutrition which impairs the immune response and predispose to invasive infection. Comorbidity of diarrhea with malnutrition makes the child more vulnerable to invasive infection [11, 18]. A number of previous studies [19–21] have been shown to have significant association of severe acute malnutrition with* Klebsiella *bacteremia.

Abnormal mentation as observed in this study was an associated factor with* Klebsiella *bacteremia which may also be common in any bacteremia and might be due to compromised perfusion or systemic response to* Klebsiella *bacteremia which is consistent with other studies with any bacteremia [22].

The main limitation is the retrospective nature of the study which involves a small number of samples that limited the power of the study and subsequently limited the generalisability of the study results. Another limitation is the lack of specification of* Klebsiella* species into *K*. pneumoniae and* K*.* oxytoca*.

In conclusion, the prevalence of* Klebsiella *bacteremia in diarrheal children was less than 1%. Diarrheal children under five with* Klebsiella *bacteremia had greater case fatality rate compared to those without bacteremia. The study highlights the importance of obtaining blood cultures in hospitalized children under five years old with diarrheal illness in the presence of either infancy, abnormal mentation, severe wasting, severe dehydration, hospital-acquired infection, or positive stool culture, although hospital-acquired infection and positive stool culture were identified as the independent risk factors for* Klebsiella *bacteremia in our study population. Awareness and identification of these simple clinical characteristics may help in early case detection and management of* Klebsiella *bacteremia and therefore help in reducing deaths in such children from developing countries. However, further research in diarrheal children with* Klebsiella *bacteremia with larger sample is warranted to substantiate our observation.

## Figures and Tables

**Table 1:** Characteristics of under-five diarrheal children with (cases) and without (controls) *Klebsiella* bacteremia at hospitalization and their outcome during hospitalization.

Characteristics	Culture positive *n* = 30 (%)	Culture negative *n* = 90 (%)	OR (95% CI)	*p*
Young age (<12 months)	24 (80)	53 (59)	2.79 (1.00–8.5)	0.037
Male	19 (63)	53 (60)	1.14 (0.45–2.93)	0.933
Duration of diarrhea in days (median IQR)	4 (2.00–7.00)	3 (2.00–5.00)	—	0.193
Antibiotics prior to admission	28 (97)	67 (78)	0.13 (0.01–0.97)	0.05
Severe dehydration	6 (20)	4 (5)	5.25 (1.18–24.56)	0.017
Fever (≥38°C)	8 (27)	47 (53)	0.32 (0.14–0.87)	0.023
Abnormal mentation	25 (83)	41 (57)	5.48 (1.92–15.67)	<0.001
Systolic BP (mean ± SD)	86 ± 13	93 ± 19	—	0.218
Diastolic BP (mean ± SD)	53 ± 11	55 ± 14	—	0.799
Hypotension	3/17 (18)	5/29 (17)	1.03 (0.16–6.15)	1.00
Fast breathing	9 (30)	46 (52)	0.4 (0.15–1.05)	0.064
SpO_2_ (mean ± SD)	96.70 ± 3.08	96.56 ± 3.41	—	0.847
Creatinine (mean ± SD)	49.80 ± 39.59	42.80 ± 45.91	—	0.250
Severe wasting	13 (59)	19 (26)	4.18 (1.39–12.81)	0.007
Severe underweight	21 (72)	46 (55)	2.16 (0.8–6.04)	0.151
*Vibrio cholerae*	1 (3)	3 (3)	0.33 (0.01–3.92)	0.614
*Shigella*	5 (17)	5 (6)	0.34 (0.08–1.4)	0.132
Positive stool culture	13 (43)	10 (11)	6.12 (2.08–18.28)	<0.001
HAI	11 (37)	2 (2)	25.47 (5.21–124.43)	<0.001
Death	12 (40)	3 (3)	19.33 (4.41–97.31)	<0.001

Figures represent *n* (total number), unless specified. OR: odds ratio. CI: confidence interval. IQR: interquartile range. SD: standard deviation. SpO_2_: transcutaneously measured blood oxygen concentration. HAI: hospital acquired infection.

**Table 2:** Results of logistic regression analysis to explore the independent association of *Klebsiella *bacteremia in diarrheal children under five years of age.

Characteristics	OR	95% CI	*p*
Young age (<12 months)	4.72	0.06–349.48	0.479
Severe wasting	10.26	0.57–185.56	0.115
Positive stool culture	26.51	1.05–671.23	0.047
Abnormal mentation	0.36	0.005–24.54	0.633
Severe dehydration	24.15	0.44–1310.45	0.118
HAI	307.1	2.16–43658.91	0.024
Hypotension	20.50	0.39–1073.92	0.135
Fast breathing	0.02	0.00–1.47	0.075

**Table 3:** Bacterial isolates from stool culture.

Organism isolated	Cases (*n* = 13) (%)	Controls (*n* = 10) (%)
*Vibrio cholerae*	1 (3)	3 (3)
*Shigella *species	5 (17)	5 (6)
*Salmonella typhi*	0	1 (1)
Other vibrios	2 (7)	1 (1)
Other organisms	5 (17)	0
